# Factor Structure and Reliability of the Revised Dyadic Adjustment Scale (RDAS) in Iranian Population

**Published:** 2012

**Authors:** Omid Isanezhad, Seid-Ahmad Ahmadi, Fatemeh Bahrami, Iran Baghban-Cichani, Ziba Farajzadegan, Ozra Etemadi

**Affiliations:** 1 Department of Counseling, Faculty of Education and Psychology, University of Isfahan, Isfahan, Iran.; 2 Department of Community Medicine, Medical University of Isfahan, Isfahan, Iran

**Keywords:** Factor Structure, Marital adjustment, Marital Happiness, Marital Satisfaction, Persian Reliability, Revised Dyadic Adjustment Scale

## Abstract

**Objective: **To determine the reliability and validity of the Revised Dyadic Adjustment Scale (RDAS) with 14 items (short form) in Iranian population.

**Methods:**The English version of the RDAS was translated into Persian. Then, Persian version was retranslated to English. To study factor structure 338 questionnaires were filled out by parents of elementary students. Other measurement tools were The Marital Happiness Scale (MHS), Enrich Marital Satisfaction Questionnaire (ENRICH).Both English and Persian forms were completed by 35 married undergraduate English students. The Cronbach's alpha coefficients of the RDAS, MHS, and ENRICH were 0.79, 0.87. and 0.76-0.91, respectively.

**Results**: The content validity of the backward translation of the original version was confirmed.The findings confirmed the factor structure.Also, the validity was confirmed by retest and internal consistency. There was relationship between RDAS with marital happiness and satisfaction in anticipated direct, between husband and wifeadjustment scores.

**Conclusion:** The reliability and validity of the RDAS with 3-factor structure in Iranian population was confirmed with an appropriate validity and reliability.

## Introduction

Previous studies have confirmed the effect of marital relationship quality on physical and mental health, and on social- mental adjustment in different dimensions of social and personal life(1-3).Marital adjustment is an important factor of family mental system(4,5).Marital adjustmenthas been developed and extended in an effort to move from person-center to familial and maritalconcepts (6,7).Marital adjustment is a process which is more than being a trait or behavior. It is an indicator of the rate of couple's adaptation in relationship with each other(8,9). This indicates general adaptation and consistency of couple’s behavior in the family and marital relationship frame and is not necessarily similar with satisfaction.It is possible that couples have adjustment but do not have satisfaction regarding their current situation. It is also likely that couples express satisfaction regarding their marital relationship quality inspite of inconsistency, maladaptation and maladjustment between their behaviors(10,11).In spite of important role ofmarital adjustmentin life and its significant contribution to determine marital health and wellbeing(9,12,13), one of the most important problems of researchers and therapists involved in family counseling is to access to an appropriate measure for assessing marital adjustment(14-16).

Marital adjustment is necessary for accessing to a functional and consistent marital relationship (17-19). The adjusted relationship is defined as a relationship in which even if the partners oppose each other, they make a good relationship and solve their common problems in a satisfied and mutual way (20). Spinier defined adjustment as a process that its consequences can be identified with the rate of couples’ problematic conflicts, interpersonal tensions, individual anxiety, marital satisfaction, coherence integrity, and collaboration about marital important problems(21,22).So, marital adjustment is a concept of multiple components that consider not only individual but also relationship with his/her partner (23, 24). 

Spanierin 1974, developed Dyadic Adjustment Scale (DAS) for assessing marital adjustment and ithas been accepted by researchers and clinicians (25-27). With revised and extension of the researches related to adjustment simultaneously with extension of related literature around this construct, Spanier's marital DAS has been revised and 1-, 6-, 7-, and 14-item forms have been developed(28-31). The 14-items formRDAS have been used for assessing marital adjustment as well as therapeutic and intervention consequences (10,29,32). This questionnaire has 3 first order factors that include consensus, satisfaction, and cohesion (28). The results of confirmatory factor analysis in previousresearcheshave confirmed 3 factors structure that have been provided by Busby et al (15, 28, 29).

The marital adjustment and its derived measures are the most famous and usefulness tools in the family and marital field in the world and have been used in thousands of studies in several languages. However, there is no study about validation and structure of this scale in Iranian population.

The current study aimed to determine the reliability and validity of revised short- form (14 items) of RDAS that developed by Busby et al. (28).

## Materials and Methods

Thisstudy was conducted in two stages. Firstly, RDAS was translated into Persian. The translated version and the original RDAS were matched and reliability of the Persian form was assessed in a preliminary sample (N=35). Next, confirmatory factor analysis (CFA) was used to assess the factor structure of RDAS (N=338). Then, the relationships between scores of RDAS, marital satisfaction and marital happiness were obtained.

*Translation*

The Englishversion of the RDAS was translated into Persian and confirmed by three bi- lingual exports in marital therapy. After that Persianversion was retranslated to English andfive English experts confirmed the backward translation. Both English and Persian formswere completed by 35 married subjects (25 females, 10 males) who were undergraduate students in English language. They were randomly selected from married undergraduate students in English language department in Isfahan University.

The rate of matching between two forms was assessed by correlation between two forms (0.91, p<0.001). Also, they scored the rate of matching between two forms in Likert scale from 5 (totally matched) to 1(totally mismatched). Twenty-onestudents scored the rate of matching as totally matched and 14 students scored them as somehow matched. The reliability in this sample attained by Cronbach's alpha coefficient was 0.79.

*The study of factor structure*

The participants were 400 people (200 couples) who were randomly selected from the parents of elementary students in Isfahan by cluster sampling. Of these, 375 questionnaires were sent back to us, but just 338 questionnaires were filled out. These included 162 couples and 14 females whose husbands were not accessible at the time of questionnaire completion. Mean (±SD) age of females and males were 26.26 (6.91) and 33.14 (7.11) years, respectively. 

Measurement tools were three questionnaires as follows:

*Revised Dyadic Adjustment Scale (RADS)*

This scale was developed by Busby et al(28).The original version was developed by Spanier in1976 according to histheory about quality of marital relationship (21). Bradbury, Fincham and Beach introduced this scale for assessing quality of marital relationship (17). This 14-item questionnaire was developed using 32 items ofthe original form which was invented by Spanier and include Likert scale. This questionnaire includes three subscaleswhich are collaboration, consensus, satisfaction and coherence that totally show marital adjustment. Higher scoresindicate better marital adjustment (29). The confirmatory factor analysis in the UShas already confirmed the 3 factor structure and its validity (28,33).The Cronbach's alpha coefficients in previous studieshave been reportedfrom 0.80 to 0.90(29).In the current study the reliability in the preliminary sample (N= 35) was 0.79.

*Marital Happiness Scale (MHS)*

This scale was created to assess marital happiness. The 10 items scale was initially devised to test the mutual observation effect which is a behavioral approach in marital counseling. Each item can be used as an independent index for marital happiness in a special domain of marital interaction. The total score of items is used to obtain marital happiness total index. It uses Likert scale with 10 scores (from 1 to 10) that respondentsscore it regarding to the rate of their happiness in each item (34, 35).

**Table .1:** Mean, standard deviation and internal

**Variables**	**1**	**2**	**3**	**4**
**Cohesion**	1			
**Consensus**	0.36	1		
**Satisfaction**	0.34	0.59	1	
**Total score**	0.69	0.61	0.24	1
**Mean**	9.05	15.41	12.16	36.63
**Standard Deviation**	3.32	4.36	3.29	8.73

correlation of the factors of RDAS

N=338                    P<0.05

Former studies have demonstrated this scale to be sensitiveenough to assess changes. This scale has significant correlation with Lock-Wallace marital adjustment test (LWMAT) (36). The Cronbach's alpha coefficient in previous studies were computed from 0.90 (38) to 0.90(34).In the current study the reliability in the preliminary sample (N= 35) was 0.87.

*Enrich Marital Satisfaction Questionnaire (ENRICH)*

This questionnaire has 115 items used for assessing problematic domains or powerful points of marital relationship (37).It scores in Likert scale from 4 (very much) to 0 (very little). The Cronbach’s alpha coefficients for each itemhave been reported from 0.47 to 0.91 by itsconstructors. In the current study, the reliability in the preliminary sample (N= 35) was obtainedfrom 0.76 to 0.91.

CFA was used to estimate the conceptual model that presented by constructors of RDAS, and Lisrel8.8 was used to assess the model fitting indices. The indices that should be stated in reporting of goodness of fit statistics include the chi-square, root mean square of approximation (RMSEA) and goodness of fit index (GFI). These statistics provide a comprehensive assessment of the fit of the model to the data (38). The chi-square statistic is mainly an important goodness of fit statistic in small samples, making it less useful in this study. Scores of less than 0.05 for the RMSEA and standardized root mean square residual (SRMR) are considered good fitting models, and 0.08 is considered as anadequate value. The RMSEA is an especially important statistic with larger samples (38, 39). The GFI, adjusted goodness of fit index (AGFI) and comparative fit index(CFI) indicated goodness of fit with scores of .90 and higher(39).

## Results

The reliability of marital adjustment was obtained using internal consistency (Cronbach’s alpha coefficient) and retest. The Cronbach’s alpha coefficient of marital adjustment scale in a sample with 338 individuals was 0.86. Also, the correlation between each item and the total score was from 0.81 to 0.88 which is indicator for competency of all items of the questionnaire. The retest administrated during 14 to 20 days for 338 individuals was completed by only 146 couples.The correlation of test and retest in two steps was 0.71.

The mean, standard deviation and internal correlations of the factors of marital adjustment scale are presented in table 1.

There were positive correlations between all of the subscales.The lowest correlation related to satisfaction factor with total score (0.24) and the highest correlation was between cohesion with total score of marital adjustment (0.69).

The result of confirmatory factor analysis according to constructors’ modelispresented in table 2.

**Table 2:** Goodness of fit indexes of confirmatory factor analysis

**CFI**	**SRMR**	**AGFI**	**GFI**	**RMSEA**	**df**	**X2**
1.00	0.28	0.99	0.99	0.001	74	28.59

As presented in table 2, RMSEA was less than 0.05 and GFI was higher than 0.90 and showed that the model has enough fitness, homogenous with data, and confirmed three-factor structure of the scale.

**Figure 1:**
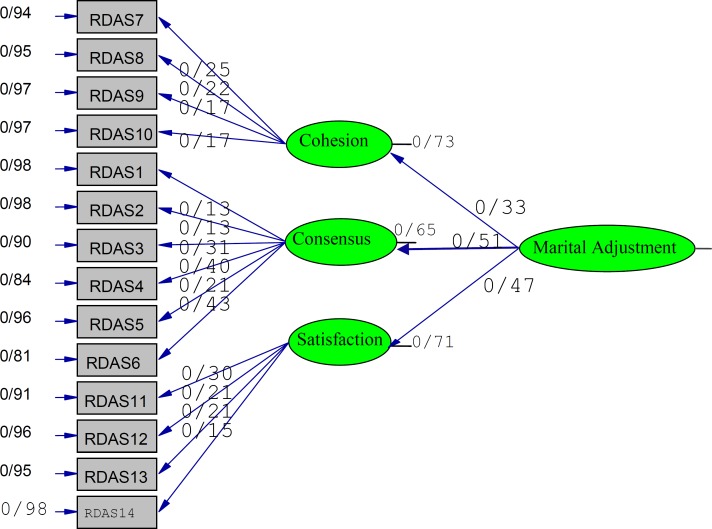
Model and standard regression coefficients of RDAS

Figure 1 shows the model and standard coefficient.The consensus subscale has higher factor load on marital adjustment.The T values for each path are higher than 1.96 (P<0.05). The three-factor structure of the RDAS was confirmed. 

**Table 3:** Correlation matrix of RDAS, MHS and ENRICH

**variable**	**1**	**2**	**3**	**4**	**5**
**ENRICH** †	1				
**MHS** ‡	0.34	1			
**Total score (RDAS** § **)**	0.52	0.61	1		
**Cohesion (RDAS)**	0.52	0.48	0.80	1	
**Consensus (RDAS)**	0.31	0.49	0.86	0.54	1
**Satisfaction (RDAS)**	0.54	0.61	0.54	0.48	0.46

*N = 338, P < 0.01, *

**†** *ENRICH Marital satisfaction scale,*

**‡** *Marital happiness scale,*

**§** *Revised dyadic adjustment scale*

To determine validity of the RDAS, its correlation with marital happiness and marital satisfaction scores were assessed (Table 3). There were statistically significant correlations between these three scales (P <0.01).

The correlation of spouse score (adjustment score) of 162 wives with 162 husbands was 0.86(P<0.01).

To study the gender differences in the RDAS values, multivariate analysis of variances (MANOVA) was used. The results of Willks’ Lambda showed that there were no significant differences betweenthe two genders in the RDAS and its components (p> 0.05).

**Table 4:** Results of MANOVA to compare the two genders in RDAS

**Value F**	**Hypothesis df**	**Error df**	**Sig.**	**Partial Eta Squared**	**Observed Power**
.99 .75	4	333	.55	.009	24

## Discussion

The researchers and clinicians, who are interested in measuring adjustment, frequently confront with some limitations for selecting an appropriate instrument.The current instruments, due to being long (such as DAS), or assessing one dimension (such as LWMAT) or no agreement between their items with current theories (such as DAS), do not seem to be practically efficient. The RDAS has have been created to compensate these limitations. 

This study aimed to determine factor structure and validation of the RDAS to be used in Iranian population.The results showed that both English and Persian forms were consistent and the subjects had similar perception towards both questionnaire forms. 

The factor structure was confirmed. So, the presented model by Busby et al (28) was confirmed in Iranian population. These findings are consistent with pervious findings about factor structure of the RDAS(28-30). It is notable that the kind of items and their contents are focused on special behavior not on attitude or belief.

The findings showed the positive correlation between the RDAS and its dimensions with the ENRICH. This correlation is an indicator for validity. This finding is compatible with previous studies (34). Also, the positive correlation between the RDAS with the MHS showed the validity of the RDAS. According to previous studies marital adjustment, satisfaction, happiness made the quality of marital relationship.So, it can be expected that increasing adjustment and adherence in marital relationship lead to increase in the marital happiness and satisfaction (24).

The findings showed the correlation between reported adjustment rate by person and his/her spouse's adjustment scores. Since marital adjustment is an interpersonal issue and items of the RDAS are focused on special and concreteness behaviors, it is supposed that there is positive correlation between spouse's marital adjustment and therefore is assumed to be an indicator for scale validity. The RDAS has satisfactory content validity. As a result, marital adjustment scale promise as a measure of marital adjustment in Iranian population.

Being a behavioral scale is an advantage of the RDAS which makes it suitable for interventions such as treatment and training (10). 

It is notable that the RDAS is particular for couple interpersonal relationship and its usage for other purposes should be done with more cautious. This is mainly due to differences between the nature of interpersonal issuesin a marriage and other extramarital relationships. 

In conclusion, the findings showed that the RDAS with 3-factor structure has appropriate validity and reliability and can be used in clinical and researches affairs in Iranian population.

## Authors' contributions

OI is a PhD student and heconceived and designed the evaluation and helped to draft the manuscript. SAA participated in designing the evaluation and performed parts of the statistical analysis. FB re-evaluated the clinical data, revised the manuscript and performed the statistical analysis and revised the manuscript. IBC translated the scale; OI collected the clinical data, AF interpreted data and revised the manuscript. OE re-analyzed the clinical data and revised the manuscript. All authors read and approved the final manuscript.

## Notes

**Declaration of Interests: **None.
